# High temperatures and human pressures interact to influence mortality in an African carnivore

**DOI:** 10.1002/ece3.7601

**Published:** 2021-06-04

**Authors:** Daniella Rabaiotti, Rosemary Groom, J. Weldon McNutt, Jessica Watermeyer, Helen M. K. O'Neill, Rosie Woodroffe

**Affiliations:** ^1^ Institute of Zoology Zoological Society of London London UK; ^2^ Division of Biosciences Department of Genetics, Evolution and Environment Centre for Biodiversity and Environment Research University College London London UK; ^3^ African Wildlife Conservation Fund Chishakwe Ranch Zimbabwe; ^4^ Botswana Predator Conservation Trust Maun Botswana; ^5^ Durrell Institute of Conservation and Ecology School of Anthropology and Conservation University of Kent Kent UK

**Keywords:** climate change, human–wildlife conflict, *Lycaon pictus*, mortality, survival, temperature

## Abstract

1. The impacts of high ambient temperatures on mortality in humans and domestic animals are well‐understood. However much less is known about how hot weather affects mortality in wild animals. High ambient temperatures have been associated with African wild dog *Lycaon pictus* pup mortality, suggesting that high temperatures might also be linked to high adult mortality.
2. We analyzed mortality patterns in African wild dogs radio‐collared in Kenya (0°N), Botswana (20°S), and Zimbabwe (20°S), to examine whether ambient temperature was associated with adult mortality.
3. We found that high ambient temperatures were associated with increased adult wild dog mortality at the Kenya site, and there was some evidence for temperature associations with mortality at the Botswana and Zimbabwe sites.
4. At the Kenya study site, which had the highest human impact, high ambient temperatures were associated with increased risks of wild dogs being killed by people, and by domestic dog diseases. In contrast, temperature was not associated with the risk of snare‐related mortality at the Zimbabwe site, which had the second‐highest human impact. Causes of death varied markedly between sites.
5. Pack size was positively associated with survival at all three sites.
6. These findings suggest that while climate change may not lead to new causes of mortality, rising temperatures may exacerbate existing anthropogenic threats to this endangered species, with implications for conservation. This evidence suggests that temperature‐related mortality, including interactions between temperature and other anthropogenic threats, should be investigated in a greater number of species to understand and mitigate likely impacts of climate change.

​

## Graphical abstract

We analyzed mortality in African wild dogs across three sites with differing human pressures. We show that at one site there is an impact of high temperatures on African wild dog mortality and that temperature and human pressure can interact to impact African wild dog mortality. These findings suggest that rising temperatures may exacerbate existing anthropogenic threats to this endangered species.

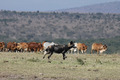

## 1 INTRODUCTION

Weather conditions have well‐documented impacts on mortality in both humans and domestic animals. Mortality rates in humans have been found to increase by 1%–3% per °C above site‐specific temperature thresholds across many parts of the globe (Hajat & Kosatky, 2010). Much of this increased mortality at high temperatures is attributed to increased risk of cardiovascular, respiratory, and cerebrovascular disease. However, death rates due to many other diseases have also been found to increase at high ambient temperatures (Basu & Samet, 2002). It has been widely acknowledged that the negative impacts of high temperatures on human mortality are likely to increase under climate change (Barros et al., 2014; IPCC, 2014). Increased mortality rates at higher temperatures have likewise been documented for domestic animals, including chickens (Warriss et al., 2005), cattle (Cox et al., 2016), and pigs (D'Allaire et al., 1996). As in humans, there is evidence that such increases in mortality for some species are caused by the interaction between heat and other forms of disease, as opposed to heat stress directly (Cox et al., 2016; D'Allaire et al., 1996). While correlations between mortality rates and ambient temperature are well‐documented in humans and domestic animals, and some of the mechanisms driving the increase in human mortality in hot weather are well‐understood, much less is known about how wild animal mortality rates might be impacted.

Extreme weather events, such as heatwaves, regularly cause mass die‐offs in wild mammals and birds (Gordon et al., 1988; Jones et al., 2018; Welbergen et al., 2008). Less well studied, however, are the impacts of increasing average daily temperatures on mortality rates of wild species outside of such extreme weather events. A wide variety of species have been documented changing their behavior in response to warmer temperatures (Briscoe et al., 2014; Hetem et al., 2012; Martin et al., 2015), and such changes may entail trade‐offs between behavioral thermoregulation and selection of favored or optimal habitats (Farmer & Brooks, 2012; Pigeon et al., 2016) or foraging success (Cunningham et al., 2015). These behavior changes have been linked to lower recruitment in a number of taxa, including birds (Cunningham et al., 2013; Nord & Nilsson, 2016; Sillett et al., 2000), mammals (Koons et al., 2012; Woodroffe et al., 2017), fish (Bogstad et al., 2013), and reptiles (Schwanz et al., 2010). Changes in species behavior in response to high temperatures have also been shown to impact adult mortality, particularly in cases where behavioral changes bring animals into closer contact with human threats, and experience higher mortality as a result. The best studied example of this phenomenon is roadkill rates in reptiles, which have been found to increase at higher temperatures in a number of studies due to higher activity levels at higher temperatures (Farmer & Brooks, 2012; Shepard et al., 2008).

**FIGURE 1:**
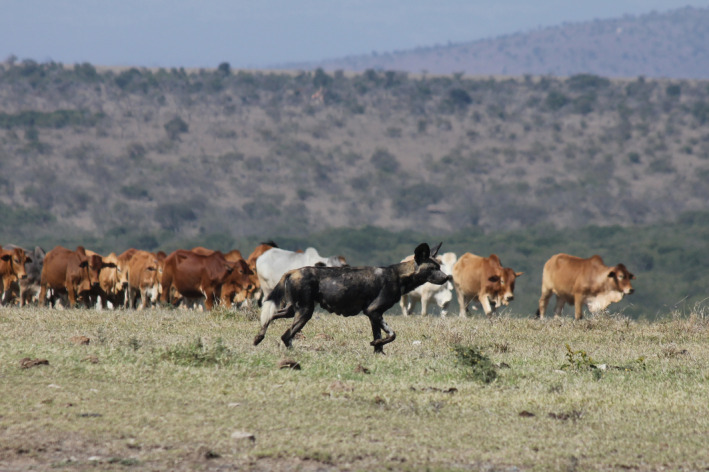
An African wild dog (*Lycaon pictus*) passes a herd of cows in Laikipia, Kenya. Photo: Helen O'Neill

One species that has been found to change its behavior at high temperatures is the African wild dog, *Lycaon pictus* (Figure 1). The African wild dog is a highly social species of canid that historically lived throughout much of sub‐Saharan Africa; however, today the species is restricted to just 7% of its historic range (Woodroffe & Sillero‐Zubiri, 2012). The main threats to the species include habitat loss, accidental snaring, intentional killing by people, and diseases acquired from domestic dogs (Prager et al., 2012; Woodroffe & Sillero‐Zubiri, 2012). These threats vary between sites (Woodroffe, Davies‐Mostert, et al., 2007), but are all related to human expansion into wild dog habitat. African wild dogs' social behavior means that human killings can result in disproportionate impacts at a population level as, if one of the dominant pair dies, the pack will often splinter, meaning that the death of one individual can reduce survival and recruitment rates for the remaining animals (Woodroffe, O'Neill, et al., 2019; Woodroffe, Rabaiotti, et al., 2019). Sociality is important for African wild dog hunting, reproduction, and defense against inter‐ and intraspecific competitors, and larger packs have consistently greater reproductive success, in terms of both litter size (Creel et al., 2004; Rasmussen et al., 2008; Woodroffe et al., 2017) and the production of daughter packs (Woodroffe, O'Neill, et al., 2019).

High ambient temperatures have been shown to influence both African wild dog behavior and reproductive success. African wild dogs are crepuscular, hunting at dawn and dusk when ambient temperatures are low (Woodroffe et al., 2017), and avoiding the heat of the day by resting in the shade. On hot days, African wild dogs are less active and travel less far, restricting their morning and evening hunts to shorter time periods (Pomilia et al., 2015; Rabaiotti & Woodroffe, 2019; Woodroffe et al., 2017). They also spend more time active at night following hot days, which may bring them into greater risk of contact with predators and of interspecific competition (Rabaiotti & Woodroffe, 2019). African wild dog birth timing is linked to ambient temperature, with packs at latitudes >2° rearing pups at the coolest time of year (McNutt et al., 2019), and those at the lowest latitudes showing longer interbirth intervals when they reared their previous litters at higher ambient temperatures (Woodroffe et al., 2017). High temperatures during African wild dog pup rearing are also associated with lower pup survival (Woodroffe et al., 2017). This impact on recruitment may reflect decreased food provisioning, and/or reduced pup guarding during periods of hot weather when adult food intake is likely to be low (Woodroffe et al., 2017).

As high ambient temperatures affect the hunting behavior of adult African wild dogs (Rabaiotti & Woodroffe, 2019), and the survival of their pups (Woodroffe, et al. 2017), and because high temperatures have been linked to increased mortality in humans and domestic animals, we predicted that high ambient temperatures would also be associated with increased adult mortality in African wild dogs. We tested this hypothesis by investigating variables associated with adult mortality in African wild dogs at three sites, representing a range of environmental conditions.

## 2 METHODS

We investigated the relationship between human activity, temperature, and wild dog mortality by (a) examining the mortality rates and causes of wild dogs at three sites of varying human impact and (b) identifying variables that correlate with wild dog mortality at each site.

### 2.1 Study sites

We analyzed African wild dog mortality at three sites: the Ewaso ecosystem, Kenya; the Okavango Delta, Botswana; and Savé Valley Conservancy, Zimbabwe. All three study sites fall within semi‐arid savanna ecosystems. However, there are marked differences between the sites in human activities and climatic conditions.

#### 2.1.1 Kenya study site

The Kenya study site (37°2′E, 0°6′N, 1,800 m ASL) covers Laikipia County, incorporating parts of neighboring Samburu, Isiolo, and Baringo counties. Relative to the other two sites, it is a high human impact area with a mix of privately owned ranches and community land. Primary land uses are subsistence pastoralism, cattle ranching, and wildlife‐based tourism. Lion density at the site is estimated at 5/100 km^2^ and is considered depressed primarily due to conflict with livestock farmers (Woodroffe & Frank, 2005). The region has short, irregular wet and dry seasons and low levels of seasonality (Franz et al., 2010). At a weather station within the study site, daily maximum temperatures ranged from 21 to 39°C (Caylor et al., 2017) throughout the data collection period of 2001 to 2016. During the same period, mean annual rainfall was 590 mm (Caylor et al., 2017).

#### 2.1.2 Botswana study site

The Botswana study site (23°38′E, 19°30′S, 960 m ASL) includes sections of the Moremi Game Reserve and adjacent Wildlife Management Areas on the eastern side of the Okavango Delta. Relative to the other sites, it is a very low human impact site—Moremi is a nationally protected area with no human habitation, surrounded by community land, which is managed for wildlife. The area comprises savanna woodland and seasonal floodplains. Lion density across the Okavango Delta varies dependent on habitat but averages 5.8/100 km^2^, with very low densities (<1/100 km^2^) in the interior of the protected area rising to 23.1/100 km^2^ in the floodplains where much of the study was done (Cozzi et al., 2013). Most rain falls during a single annual rainy season between November and March, coinciding with the hottest part of the year. The nearest weather station is 30 km outside the study site at Maun airport. Mean annual rainfall over the data collection period from 1992 to 2004 was 422 mm. Maximum daily temperatures ranged between 23 and 40°C in the hot season (months September–December) and 20 and 35°C degrees in the cool season (months June–August).

#### 2.1.3 Zimbabwe study area

The Zimbabwe study site (32°00′E, 20°05′S, 550 m ASL) was the Savé Valley Conservancy in the South Eastern Lowveld. The site has intermediate human impact relative to the other two sites, being comprised of contiguous private land managed for wildlife, surrounded by community land, which is not managed for wildlife. The area is primarily woodland savanna covering low hills, interspersed with rocky outcrops. Like the Botswana site, the Zimbabwe site experiences a single warm, wet season each year, with most rain falling between November and March. Weather data were obtained from the Middle Sabi Research Station, 12 km from the study area boundary. Throughout the data collection period, from 2008 to 2017, daily maximum temperatures ranged between 22 and 48°C in the hot season (months October–December) and 17–38°C in the cool season (months June–August). Mean annual rainfall was 381 mm.

### 2.2 Field data collection

At the Kenya study site, 130 African wild dogs (56 female and 74 male) from 41 packs were monitored using either Vectronics GPS collars (GPS Plus; Vectronic Aerospace GmbH), Televilt GPS collars (GPS‐Posrec; Televilt), or VHF radio collars (Telonics). All three collar types included a mortality sensor programmed to emit a characteristic radio signal if stationary for ≥4 hr. At the Zimbabwe study site, 59 African wild dogs (22 female and 37 male) from 34 packs were monitored using either radio collars or GPS collars (African Wildlife Tracking). Using radio collars (Sirtrack), 31 African wild dogs (10 female and 21 male) from 16 packs were monitored at the Botswana site. Collars were fitted using the procedures outlined in McNutt (1996), Woodroffe (2011), and Jackson et al. (2017).

At all three sites, packs were located every 1–2 weeks where possible. Any collared animal found dead was carefully examined with the aim of establishing a cause of death. At the Kenya site, necropsies were carried out on all dead individuals located. At the Botswana site, cause of death was only recorded in cases where the death was directly observed, or during disease outbreaks, and therefore, the majority of causes of death were unconfirmed. Most deaths at the Botswana site are likely to be due to natural causes given the low human activity in this area. For all three sites, the date of first detection of a mortality signal from the collar was used to estimate the date of death when not observed directly, and where this was not possible, an estimated date of mortality was made based on the date midway between the last sighting, or the last detection of the radio collar without a mortality signal, and the discovery of the carcass or collar. If any study animal was not observed in its resident pack for over 30 days, no mortality signal was detected, and no carcass was found, it was considered lost from the study and censored from the day of the last observation (Kenya: *n* = 51, Zimbabwe: *n* = 34, Botswana: *n* = 8). If a carcass or collar was discovered more than 30 days after the last sighting (*n* = 2), the animal was considered lost from the study due to the inaccuracy of the date of death and was censored from the date of the last sighting.

Group and individual characteristics were recorded at each site. At all three sites, dispersal status of the individual was recorded. Individuals were defined as dispersing if they left their pack for multiple days and did not return, otherwise they were defined as resident (Woodroffe, Rabaiotti, et al., 2019). Group size—either the pack size for resident individuals or the dispersal group size for dispersing individuals—was recorded for each individual and was defined as the number of adults (>12 months in age) in the group. African wild dog pup rearing involves the pups being left at a den site for the first 3 months of life, while the majority of the rest of the pack hunt daily, bringing food back to provision the pups. This pup rearing period is referred to as denning. For each pack, denning periods were identified using either direct observations or GPS collar data.

At the Kenya site, a number of other individual and pack characteristics were also monitored. Individuals' alpha status was inferred based on consistent close association with a specific individual of the opposite sex, coordinated scent marking, and reproductive activity; all animals not identified as alpha were considered subdominant. African wild dog age was known for many individuals, otherwise it was estimated from tooth wear when the individual was collared (Woodroffe, Rabaiotti, et al., 2019). Age range at collaring ranged from 1 to 7 years (mean: 2.43 ± 1.27). The age of the majority of individuals at the Zimbabwe and Botswana sites was not known.

### 2.3 Data analysis

#### 2.3.1 Mortality rates and causes

We divided mortality into four causes: (a) intentional killing by people (e.g., shooting); (b) unintentional killing resulting from human activity (e.g., roadkill or snaring); (c) natural causes—this category included injuries sustained while hunting, death by other predators, or intraspecific fighting; and (d) disease. Disease was separated from other causes because most diseases were associated with domestic dogs (Woodroffe et al., 2012) and therefore were arguably human‐caused. Where the cause of death could not be confidently established, the deaths were categorized as having unconfirmed cause.

Annual mortality rates for all causes of death combined were estimated using the Kaplan–Meier method from the *survival* package (Therneau, 2015; Therneau & Grambsch, 2000) in *R* (3.6.0, R Core Team, 2019).

#### 2.3.2 Correlates of mortality

For each site, we investigated the relationship between African wild dog mortality and a number of explanatory variables. Variables investigated depended both on the data available and size of the dataset at each site and for each cause of death (Table S1).

At all three sites, we included mean maximum temperature and total rainfall as candidate explanatory variables. Since high ambient temperature is associated with low pup survival (Woodroffe et al., 2017), we hypothesized that it might also reduce adult survival. Similarly, rainfall has previously been found to mediate temperature impacts, and also impact wild dog movement and demographic variables (Rabaiotti & Woodroffe, 2019; Woodroffe et al., 2017). In order to determine the most appropriate scale for the climatic variables at each site, we constructed a series of full models with temperature and rainfall as explanatory variables at 90‐day, 30‐day, and 7‐day scales. The scale of climatic variables in the model with the highest AIC value was then used in our analyses (Table S2a–c).

Likewise, analyses for all three sites included group size as a candidate explanatory variable. We have previously shown that mortality was lower in larger packs at the Kenya site (Woodroffe, O'Neill, et al., 2019), although the opposite effect has been reported at another site (Creel & Creel, 2002). To ensure there was no more than one explanatory variable per 10 mortality events, temperature and group size were the only two explanatory variables investigated at the Botswana and Zimbabwe sites, where fewer than 30 mortality events were recorded.

Primary analyses evaluated associations between candidate explanatory variables (Tables S3–S9) and mortality due to all causes. For the primary analysis at the Kenya site, the larger number of collared animals monitored, and corresponding greater number of deaths recorded, allowed us to also compare African wild dog mortality risk with a number of other demographic and environmental variables, namely age, individual status (alpha or subdominant), group status (denning resident, nondenning resident, or dispersing), and land use (community or private ranch). We included age because it has been found to influence survival in other species (e.g., Loison et al., 1999; MacNulty et al., 2009; Owens, 2002). We included individual status because dominance has been linked to higher stress and parasite load, as well as to higher levels of intraspecific conflict, in several social mammals and birds (e.g., Creel, 2005; Muehlenbein et al., 2010). Group status was included because dispersal has been previously linked to high mortality (Cozzi et al., 2020; Woodroffe, O'Neill, et al., 2019). Similarly, denning is a period characterized by high energetic demand (Woodroffe et al., 2017) and lack of mobility, putting wild dogs at risk from human activity (Jackson et al., 2014), meaning mortality might likewise be elevated. We also compared wild dog mortality rates with human land use. Packs could be classified into those residing primarily on community land (≥90% of recorded locations, Woodroffe (2011)) and those residing primarily on private ranches (≥70% of locations, Woodroffe (2011)). We predicted that individual wild dogs residing primarily on community land, where livestock, domestic dog, and human densities are higher (Woodroffe & Donnelly, 2011), would have higher mortality rates than those residing primarily on private ranches. Additionally, rainfall has previously been shown to have a negative effect on pup survival at the Kenya study site (Woodroffe et al., 2017), and therefore, we predicted similar impacts on adult survival.

We also conducted secondary analyses considering associations between explanatory variables and mortality due to specific causes that resulted in the death of 10 or more individual wild dogs at a single site. For these secondary analyses, deaths due to other causes were censored. At the Kenya site, intentional killings, natural causes, and disease all caused >10 deaths, providing a large enough sample size to analyze correlates of mortality separately. The only factor causing ≥10 deaths at the Zimbabwe site was unintentional human killings, and no single factor caused ≥10 deaths at the Botswana site. Analyses of specific mortality causes at the Kenya and Zimbabwe sites included only temperature, rainfall, and group size, with a maximum of two variables in each candidate model, as there were fewer than 30 mortality events for each cause of death.

Associations between daily adult mortality and each of the candidate explanatory variables were assessed in mixed‐effects Cox proportional hazards models using the “coxme” function in the “coxme” package (Therneau, 2015) in *R* (R Core Team, 2019). Group identity was included in the models as a random variable. The time origin in the models was the date of collaring, and the timescale was days since collaring.

Model selection was carried out using ΔAIC and model averaging (Burnham et al., 2002). For each site, a set of candidate models, including a null model containing just the random variable, was selected for each location, and models with ΔAIC of ≤2 were included in the top model set. Candidate model sets and corresponding ΔAIC values are shown in Tables S2–S8. Where more than one model had a ΔAIC value <2, model averaging was then carried out on the top set models using the “MuMIN” *R* package (Barton, 2009), other than in cases where the null model was the top model. All independent variables were tested for autocorrelation, and all were found to have correlation coefficients below 0.5.

## 3 RESULTS

### 3.1 Causes of death

The primary causes of death varied between the three sites (Table 1). In Kenya, natural causes accounted for the highest proportion of deaths in any category, accounting for 34% of all deaths. Twenty‐four percent of deaths in Kenya were due to human causes (both intentional and unintentional). The majority of human‐caused deaths (84%) in Kenya were due to intentional human killings. In Zimbabwe, the leading cause of death was unintentional human‐caused deaths, specifically snaring (40% of all deaths), with only one death from disease throughout the study. At the Botswana site, the majority of deaths were due to unconfirmed causes (61%, Table 1).

**TABLE 1:** Causes of African wild dog mortality at each study site

Cause	Number of deaths (% of total)		
	Kenya	Botswana	Zimbabwe
Natural	27 (34%)	3 (13%)	8 (32%)
Disease	20 (25%)	6 (26%)	1 (4%)
Intentional human	16 (20%)	0	0
Unintentional human	3 (4%)	0	10 (40%)
Unconfirmed	13 (16%)	14 (61%)	6 (24%)
Total	79	23	25

Note

Percentages of total deaths are indicated in brackets.

### 3.2 Mortality rates

Overall annual mortality rates differed across the three sites. The Botswana site had the lowest estimated mortality rate, with the highest mortality rates observed at the Zimbabwe site (Table 2).

**TABLE 2:** Estimated annual mortality rates of radio‐collared adult African wild dogs, estimated using the Kaplan–Meier method

Cause	Kenya			Zimbabwe			Botswana		
	Mortality rate	Lower 95% CI	Upper 95% CI	Mortality rate	Lower 95% CI	Upper 95% CI	Mortality rate	Lower 95% CI	Upper 95% CI
All	0.28	0.20	0.35	0.25	0.12	0.36	0.14	0.01	0.26
Natural	0.11	0.04	0.16	0.11	0.02	0.20			
Disease	0.08	0.03	0.13				0.08	0.01	0.17
Intentional human	0.10	0.04	0.16						
Unintentional human	0.01	0	0.03	0.09	0.01	0.16			
Unconfirmed	0.01	0	0.03	0.07	0.01	0.13	0.07	0.01	0.16

Note

Gray shading indicates there were no deaths from that cause recorded during the study period. Blue shading indicates that there were no deaths from that cause in the first 365 days, so an annual mortality rate could not be calculated using the Kaplan–Meier method.

### 3.3 Correlates of mortality

**FIGURE 2:**
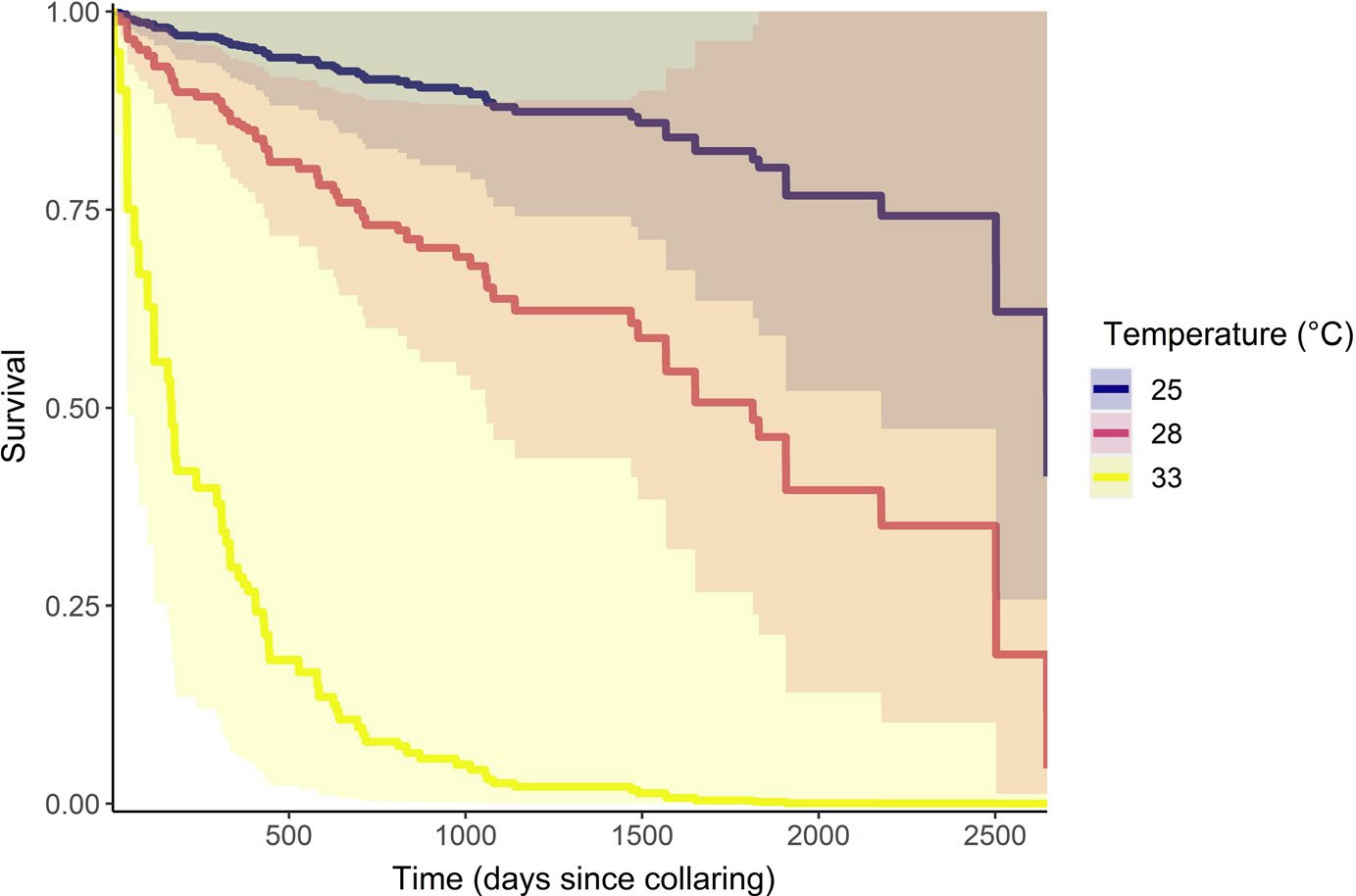
Estimated individual survival rates from mixed‐effects Cox proportional hazards models at the Kenya study site at the maximum (33°C), median (28°C), and minimum (25°C) of mean temperatures over the previous 90 days. The shaded areas represent 95% confidence intervals

**FIGURE 3:**
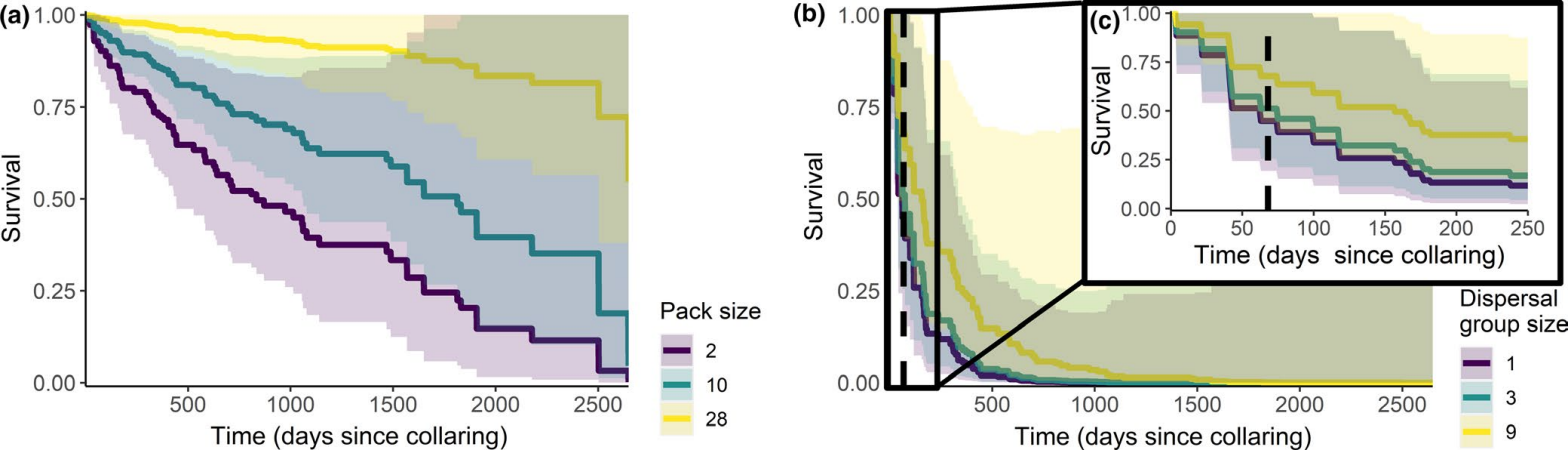
Estimated individual survival rates from mixed‐effects Cox proportional hazards models at the Kenya study site at the maximum, median, and minimum group sizes when African wild dogs were (a) resident and not denning and (b) dispersing. (c) Shows the same plot as panel b with the x‐axis truncated to 250 days. The black dashed lines on plots b and c indicate the maximum dispersal time observed in the field (Woodroffe, Rabaiotti, et al., 2019). The shaded areas represent 95% confidence intervals

Correlates of mortality are shown in Table 3. At the Kenya study site, African wild dogs had higher mortality at higher temperatures (Figure 2), when they were in smaller groups (Figure 3), when they were dispersing rather than resident (whether denning or nondenning; Figure 3), and when they were older (Table 3). There was some indication that both residing on commercial as opposed to community land and higher rainfall over the previous 30 days had a positive impact on survival; however, both 95% confidence intervals crossed zero (Table 3).

**TABLE 3:** Model‐averaged (ΔAIC < 2) results of mixed‐effects Cox proportional hazards models, considering mortality of radio‐collared animals for all causes combined

Study site	Variable	Coefficient	95% CI	Sum of weights
Kenya	Mean maximum temperature (°C, 90 days)	0.46	±0.27	1.00
	Group status: denning versus resident–nondenning	−0.077	±0.75	1.00
	Group status: dispersing versus resident–nondenning	3.12	±1.53	
	Age (days)	0.00073	±0.00073	1.00
	Dominant (yes)	−0.79	±0.74	1.00
	Group size	−0.072	±0.072	0.80
	Land use: community land versus private ranch	0.62	±0.90	0.73
	Total rainfall (mm, 30 days)	−0.0021	0.0060	0.18
Botswana	Group size	−0.18	±0.13	1.00
	Mean maximum temperature (°C, 30 days)	0.07	±0.18	0.29
	Total rainfall (mm, 7 days)	0.013	±0.024	0.23
Zimbabwe	Group size	−0.038	±0.074	0.64
	Mean maximum temperature (°C, 90 days)	−0.067	±0.21	0.50
	Total rainfall (mm, 90 days)	−0.015	±0.077	0.35
	Mean maximum temperature: Total rainfall	0.00042	±0.0023	0.14

Note

The models also include group identity as a random variable. NULL indicates that the null model was the top model in the set. Negative estimates indicate a lower probability of death, that is, higher survivorship.

When rates of mortality at the Kenya site were analyzed separately by cause of death, higher temperatures were associated with higher mortality from both disease and intentional killing by people (Table 4). There was also some indication that temperature may also be associated with mortality from natural causes; however, the 95% confidence interval crossed zero (Table 4). While pack size was in the top model set for all three causes of death, with mortality lower in larger groups, the only cause of death for which the confidence interval did not cross zero was natural causes (Table 4).

**TABLE 4:** Model‐averaged (ΔAIC < 2) results of mixed‐effects Cox proportional hazards models, considering mortality of radio‐collared animals separately for different causes

Study site	Cause of death	Variable	Coefficient	*SE*	Sum of Weights
Kenya	Human (intentional)	Mean maximum temperature (°C, 90 days)	0.82	±0.70	1.00
		Group size	−0.12	±0.14	0.52
		Total rainfall (mm, 30 days)	0.20	±0.36	0.36
			−0.011	±0.0095	0.24
	Natural causes	Group size	−0.12	±0.11	1.00
		Mean maximum temperature (°C, 90 days)	−0.12	±0.46	0.23
		Total rainfall (mm, 30 days)	−0.0030	±0.011	0.22
	Disease	Mean maximum temperature (°C, 90 days)	0.85	±0.57	1.00
		Group size	−0.029	±0.13	0.24
		Total rainfall (mm, 30 days)	0.0034	±0.011	0.23
Zimbabwe	Human (unintentional)	NULL			

Note

Models were built only for confirmed mortality causes accounting for ≥10 deaths. As there were no such causes at the Botswana site, no results are shown for this site. NULL indicates that the null model was the top model in the set. Negative estimates indicate a lower probability of death, that is, higher survivorship.

At the Botswana site, mortality rates due to all causes were lower in larger groups. There was also some support for the inclusion of temperature in the model, with higher mortality rates at higher temperatures, although the 95% confidence interval crossed zero (Table 3). At the Botswana site, no confirmed mortality causes accounted for ≥10 deaths, so mortality was not analyzed separately by cause.

At the Zimbabwe site, the null model was in the top model set, indicating that the models including the explanatory variables did not perform significantly better than the null model (Table S7). Group size, temperature, rainfall, and an interaction between temperature and rainfall were all in the top model set, with a negative relationship between both climatic variables and mortality, and a positive interaction between rainfall and temperature. There was the most support for the effect of group size, with animals in larger packs having higher survival rates. All confidence intervals crossed zero, however (Table 3). Only unintentional human causes accounted for more than 10 deaths in Zimbabwe, and therefore, this was the only cause of death for which separate models were built. The null model was also the only model with a ΔAIC of <2 when only deaths from human causes were included in the model (Table 4).

## 4 DISCUSSION

Our findings suggest that human impacts on African wild dog mortality are likely to be pervasive. When the effects of intentional and unintentional killing are combined with the effects of domestic dog diseases, 44% of deaths in this study were linked to human activity. This human‐caused mortality is likely to occur in addition to natural mortality, as overall mortality rate was markedly lower in the low human impact site in Botswana than at the more human‐impacted sites in Kenya and Zimbabwe (Table 2). As the climate warms, human impacts on wild dog populations are likely to extend beyond the immediate effects of people within wild dog range, to impacts of the global human population, via carbon emissions to the atmosphere. High ambient temperatures were associated with elevated African wild dog mortality across two out of three sites, with human activity appearing to contribute to increased mortality at high temperatures at the Kenya site, where human impacts were highest, through increased deaths due to intentional killing and disease after periods of warmer weather. At the Zimbabwe site, where a high proportion (40%) of deaths were due to unintentional human killing, specifically snaring, there was little support for a relationship between temperature and mortality, likely due to the indiscriminate nature of snaring incidents masking any relationship with the modeled explanatory variables.

Group size was associated with lower mortality rates at two of the three sites although, similar to temperature, no association was found at the Zimbabwe site, where a high proportion of deaths were due to snaring. The harmful effect of small group size on survival was especially marked for natural mortality causes (Tables 3 and 4). The findings of lower mortality in larger groups contrast with observations by Creel and Creel (2002) and Creel et al. (2004) who suggested that individual mortality (among all study animals, not just those with tracking collars) was higher in larger packs. However, a recent analysis (using data from our Botswana site) showed that a high proportion of uncollared animals recorded as missing from study packs and assumed dead are likely to have dispersed (Behr et al., 2020). This challenge of distinguishing death from dispersal may explain the discrepancy between our findings and those of Creel and Creel (2002) and Creel et al. (2004), highlighting the difficulty of exploring rates and causes of mortality from study animals without radio collars (Woodroffe, Davies‐Mostert, et al., 2007; Woodroffe, Frank, et al., 2007).

The higher rates of mortality observed at higher ambient temperatures at the Kenya site appear to reflect an increase in deaths linked to human activity; in hot weather, wild dogs were more likely to be killed (intentionally) by people, and to die from domestic dog diseases, but there was only weak support for increased mortality at higher temperatures from natural causes (Table 4). As observed in people (Basu & Samet, 2002), rather than hot weather directly leading to heatstroke and death, high temperatures appeared to exacerbate the risk of mortality from other causes. At high ambient temperatures, both people and wildlife change their behavior. In African wild dogs, high ambient temperatures have been linked to shifts in the timing of hunts and changes in habitat use (Rabaiotti & Woodroffe, 2019; Rabaiotti et al., unpublished data). During hot and dry weather, pastoralists are more likely to move with their livestock into areas further from their residences in search of grazing, and therefore, there is likely to be more overlap in habitat use between people and African wild dogs (Amphlett, 2017).

This hypothesized increased overlap in space between African wild dogs and humans would also lead to increased exposure to disease from domestic dogs and therefore increased disease levels (Woodroffe & Donnelly, 2011; Woodroffe et al., 2012). Additionally, high temperatures might also affect disease susceptibility. We have shown previously that African wild dogs appear to hunt less at high ambient temperatures (Rabaiotti & Woodroffe, 2019; Woodroffe et al., 2017), which means that, following prolonged periods of hot weather, African wild dogs are likely to be under greater energetic stress. Malnourished animals have been found to have compromised immune systems (Losada‐Barragán et al., 2017), to be more likely to contract disease (Harvell et al., 2002), and to be more likely to die once they are infected (Kim et al., 2018). Increased mortality from disease may also be driving the association between temperature and mortality at the Botswana site, as, although the majority of known‐cause deaths at the site were due to natural causes, 25% were due to disease. On the other hand, wild dogs have been found to be more active on nights following hotter days (Rabaiotti & Woodroffe, 2019), which may put them more at risk from larger predators such as lions and hyenas.

At the Kenya site, members of larger groups experienced lower mortality from all three causes that were analyzed—disease; natural causes; and human causes. The strong relationship between group size and mortality due to both natural and human causes would suggest that larger groups are better at avoiding predators, competitors, and people. Larger packs are better able to defend themselves against spotted hyenas (*Crocuta crocuta*; Fanshawe & FitzGibbon, 1993), and against other packs of African wild dogs (Creel & Creel, 2002), which is likely the driver of decreased mortality due to predators and conspecifics at higher pack sizes, as lion kills are rare at the Kenya study site. The lower individual mortality among members of larger packs due to intentional human killings may potentially occur because of the dilution effect, whereby individuals in a larger group have lower individual mortality risks (Foster & Treherne, 1981). Large packs may also be better able to support any injured members of their group (Courchamp & Macdonald, 2001). Our models suggested there may be a weak relationship between higher pack size and lower mortality from disease, which is in line with previous findings of lower seroprevalence for some pathogens in larger packs (Woodroffe et al., 2012). As larger packs consistently raise larger litters (Courchamp & Macdonald, 2001; Gusset & Macdonald, 2010), including at our three study sites (Woodroffe et al., 2017), it is likely that such packs are also more able to support a member that is unable to hunt, increasing that individual's chance of recovery from injury or disease.

Human activity had a clear impact on African wild dog mortality. Rates of mortality varied in line with human impacts—at the Kenya and Zimbabwe sites, where wild dogs had frequent opportunities to encounter human activities, the wild dog mortality caused directly or indirectly by local people exceeded that caused by all factors combined at the Botswana site, where human impacts were predicted to be low. At the Kenya site, which was both outside protected areas and had the highest human impact, African wild dogs were more likely to die on community than private lands. Community land has higher grazing pressure, lower numbers of prey species, and higher domestic dog densities than private lands (Woodroffe et al., 2005), all of which are likely to have negative impacts on African wild dogs living in the area.

The impacts of temperature varied between sites, affecting intentional human‐caused mortality and disease at the Kenya site, total mortality (most likely from natural causes) at the Botswana site, and having a weak relationship with total mortality at the Zimbabwe site. At least some of this variation appears to be driven by human activity. At the Kenya site, it appears that the higher rate of mortality at high temperatures reflects greater impacts of human activity, with higher temperatures increasing mortality rates from intentional human‐caused deaths and disease. It may be that at the Botswana and Zimbabwe sites, which experience high levels of seasonality, seasonal processes such as vegetation cover, prey densities, or timing of denning are driving the correlation between high temperatures and mortality. Due to the lower number of mortality events in these datasets, we were unable to disentangle the effects of temperature and seasonality, but since denning did not result in an increase or decrease in mortality at the aseasonal Kenya site, it seems unlikely it would be driving mortality patterns elsewhere.

Our findings of increased mortality at high temperatures raise concerns about the likely impact of climate change on this endangered species. As the climate becomes warmer, African wild dog adult mortality is likely to rise. This predicted increase in adult mortality parallels predicted falls in recruitment, as African wild dog pup survival falls at higher temperatures (Woodroffe et al., 2017). These twin effects are likely to exacerbate one another through their mutual effects on pack size. Higher adult mortality is likely to lead to smaller pack size, which in turn will lead to higher adult mortality, as well as decreased recruitment. In combination, these impacts are likely to depress both mean pack size and population size (Woodroffe, O'Neill, et al., 2019), although population modeling would be needed to quantify the specific impacts on population viability.

While the interacting effects of local human activity and global climate on African wild dog mortality are cause for concern, they also suggest that the relatively intractable threat from global climate change might be mitigated by addressing local threats. Management to resolve human–wildlife conflict and to reduce disease transmission from domestic dogs could help to make African wild dog populations more robust in the face of climate change (Dickman, 2010; Gusset et al., 2008; Prager et al., 2011, 2012; Vial et al., 2006; Woodroffe, Frank, et al., 2007).

Our results show that hot weather can increase mortality rates in wild animals; however, little attention has been given to the impact of high temperatures on wild animal mortality in other study systems. Temperatures are predicted to increase under climate change, with serious implications for the survival of temperature‐sensitive species. Thermoregulatory behaviors such as shifts in habitat use or increased time spent in thermal refugia rather than foraging come with trade‐offs (Cunningham et al., 2015; Pigeon et al., 2016), which can impact not only reproduction but also adult survival (Sinervo et al., 2010). It is important that the species that respond to high temperatures by changing their behavior in a way that reduces their ability to feed, survive, or reproduce are identified as these species are likely to be at risk from rising global temperatures.

Direct impacts of local people are likely to interact with indirect impacts of global populations for a wide variety of species. The most obvious interaction is that between climate change (caused by global carbon emissions) and habitat loss (enacted by local people), which restricts the ability of species to move to more favorable habitats as the climate warms. Our results also show, however, that other human pressures can interact with climate to influence demographic outcomes in an endangered species. Changes in habitat use, time allocation between behaviors, and timing of activity can all occur in response to high temperatures, and all have the potential to increase the overlap between wildlife and human activity, potentially leading to increases in mortality. For many species, it may be that, by reducing other human impacts, conservationists can also mitigate the impacts of climate change.

## CONFLICT OF INTEREST

None declared.

## AUTHOR CONTRIBUTION

**Daniella Rabaiotti:** Conceptualization (equal); Data curation (equal); Formal analysis (lead); Funding acquisition (equal); Investigation (lead); Methodology (lead); Project administration (equal); Resources (equal); Visualization (lead); Writing‐original draft (lead); Writing‐review & editing (lead). **Rosemary Groom:** Data curation (equal); Funding acquisition (equal); Investigation (equal); Project administration (equal); Resources (equal); Writing‐review & editing (equal). **J. Weldon McNutt:** Data curation (equal); Funding acquisition (supporting); Project administration (supporting); Resources (equal); Writing‐review & editing (supporting). **Jessica Watermeyer:** Data curation (equal); Project administration (equal); Writing‐review & editing (supporting). **Helen M. K. O'Neill:** Data curation (equal); Project administration (supporting); Writing‐review & editing (supporting). **Rosie Woodroffe:** Conceptualization (equal); Data curation (equal); Funding acquisition (equal); Project administration (equal); Resources (equal); Supervision (lead); Writing‐original draft (supporting); Writing‐review & editing (supporting).

## Supporting information

Supplementary Material

## Data Availability

All survival data and covariates are archived on Dryad https://doi.org/10.5061/dryad.4j0zpc8b9
