# Supplementary material for: High temperatures and human pressures interact to influence mortality in an African carnivore
Source: Ecol Evol. 2021 Jun 4;11(13):8495–506. doi: 10.1002/ece3.7601 (PMC8258213; doi:10.1002/ece3.7601)

## Supplementary Information

| Variables included in the candidate models for each site and cause of death. The number in brackets indicates the scale of the variable used – either the mean over that many days (temperature) or the total across that time period (rainfall). | | | | | | | | | |
| --- | --- | --- | --- | --- | --- | --- | --- | --- | --- |
| Site | Cause of Death | Group Size | Temperature | Rainfall | Temperature: Rainfall | Group status | Age | Individual status | Land Use |
| Kenya | All | x | x (90) | x(30) | x | x | x | x | x |
|  | Predator, conspecific or injury | x | x (90) | x(30) | x |  |  |  |  |
|  | Intentional human | x | x (90) | x(30) | x |  |  |  |  |
|  | Disease | x | x (90) | x(30) | x |  |  |  |  |
| Zimbabwe | All | x | x(90) | x(90) | x |  |  |  |  |
|  | Unintentional human | x | x(90) | x(90) |  |  |  |  |  |
| Botswana | All | x | x(30) | x (7) | x |  |  |  |  |

Table S1: Variables tested at each site and cause of death

Table S2a Model selection table for climatic variable scale at the Kenya site

| Temperature scale (days) | Rainfall scale (days) | AICc | ΔAIC |
| --- | --- | --- | --- |
| 90 | 30 | 396.035 | 0.000 |
| 90 | 7 | 396.372 | 0.337 |
| 90 | 90 | 396.551 | 0.516 |
| 30 | 90 | 398.314 | 2.279 |
| 30 | 7 | 398.327 | 2.292 |
| 30 | 30 | 398.333 | 2.298 |
| 7 | 90 | 402.120 | 6.085 |
| 7 | 30 | 402.150 | 6.115 |
| 7 | 7 | 402.164 | 6.128 |

Table S2b Model selection table for climatic variable scale at the Botswana site

| Temperature scale (days) | Rainfall scale (days) | AICc | ΔAIC |
| --- | --- | --- | --- |
| 30 | 7 | 87.179 | 0.000 |
| 90 | 7 | 87.425 | 0.246 |
| 7 | 7 | 87.500 | 0.321 |
| 30 | 30 | 87.832 | 0.653 |
| 30 | 90 | 87.885 | 0.706 |
| 90 | 30 | 88.085 | 0.906 |
| 90 | 90 | 88.115 | 0.935 |
| 7 | 30 | 88.193 | 1.013 |
| 7 | 90 | 88.258 | 1.079 |

Table S2c Model selection table for climatic variable scale at the Zimbabwe site

| Temperature scale (days) | Rainfall scale (days) | AICc | ΔAIC |
| --- | --- | --- | --- |
| 90 | 90 | 154.773 | 0.000 |
| 7 | 90 | 154.879 | 0.106 |
| 30 | 90 | 155.141 | 0.368 |
| 90 | 30 | 155.257 | 0.484 |
| 90 | 7 | 155.260 | 0.487 |
| 7 | 7 | 156.058 | 1.285 |
| 7 | 30 | 156.108 | 1.335 |
| 30 | 7 | 156.242 | 1.469 |
| 30 | 30 | 156.258 | 1.485 |

Table S3a: Candidate Cox Proportional Hazards models for the Kenya dataset – deaths from all causes. An “x” indicates that the variable was included in that particular model.

| Model | Group Size | Temperature | Rainfall | Temperature:Rainfall | Group status | Age | Individual status | Land Use |
| --- | --- | --- | --- | --- | --- | --- | --- | --- |
| 0 | NULL | | | | | | | |
| 1 | x | x | x |  | x | x | x | x |
| 2 | x | x | x | x | x | x | x | x |
| 3 | x | x | x | x | x |  |  | x |
| 4 | x | x | x |  | x |  |  | x |
| 5 | x | x | x | x | x | x |  | x |
| 6 | x | x | x |  | x | x |  | x |
| 7 | x | x | x | x | x |  |  |  |
| 8 | x | x | x |  | x |  |  |  |
| 9 | x | x | x | x |  |  |  | x |
| 10 | x | x | x |  |  |  |  | x |
| 11 | x | x | x | x | x |  |  | x |
| 12 | x | x | x |  | x |  |  | x |
| 13 | x | x |  |  | x |  |  | x |
| 14 | x |  | x |  | x |  |  | x |
| 15 | x | x | x |  |  |  |  |  |
| 16 | x | x |  |  |  |  |  | x |
| 17 | x |  |  |  |  |  |  |  |
| 18 | x | x |  |  |  |  |  |  |
| 19 | x |  |  |  | x |  |  |  |
| 20 | x | x |  |  | x | x | x | x |
| 21 | x | x |  |  | x | x | x |  |
| 22 |  | x |  |  | x | x | x | x |
| 23 |  | x |  |  | x | x |  | x |
| 24 |  | x |  |  | x | x |  |  |
| 25 |  | x |  |  | x | x |  |  |
| 26 | x | x |  |  | x | x |  |  |

Table S3b: Candidate Cox Proportional Hazards models for the Kenya dataset – deaths from all causes

| Model | AICc | ΔAIC |
| --- | --- | --- |
| 21 | 394.41 | 0 |
| 20 | 394.5656 | 0.155605 |
| 22 | 395.9023 | 1.492343 |
| 1 | 396.5515 | 2.141474 |
| 13 | 396.808 | 2.398007 |
| 26 | 397.3893 | 2.979329 |
| 24 | 397.4624 | 3.052458 |
| 25 | 397.6237 | 3.213768 |
| 23 | 397.693 | 3.283023 |
| 2 | 398.4724 | 4.062449 |
| 4 | 398.808 | 4.39798 |
| 8 | 399.3895 | 4.979507 |
| 6 | 399.683 | 5.273035 |
| 12 | 399.7483 | 5.338358 |
| 3 | 400.7525 | 6.342533 |
| 7 | 401.3847 | 6.974748 |
| 5 | 401.6687 | 7.258712 |
| 11 | 401.7434 | 7.333428 |
| 19 | 405.6082 | 1.12E+01 |
| 14 | 405.6611 | 1.13E+01 |
| 16 | 409.738 | 1.53E+01 |
| 15 | 411.7192 | 1.73E+01 |
| 10 | 412.8303 | 18.42033 |
| 18 | 413.5483 | 19.13837 |
| 17 | 414.45 | 20.04001 |
| 9 | 414.4648 | 20.05483 |
| 0 | 418.0988 | 23.68885 |

Table S4a: Candidate Cox Proportional Hazards models for the Kenya dataset – deaths from intentional human causes

| Model | Group Size | Temperature (90) | Rainfall (30) | Temperature:Rainfall |
| --- | --- | --- | --- | --- |
| 0 | NULL | |  |  |
| 1 |  | x |  |  |
| 2 | x |  |  |  |
| 3 | x | x |  |  |
| 4 |  |  | x |  |
| 5 | x |  | x |  |
| 6 |  | x | x |  |
| 7 |  | x | x | x |

Table S4b: Model selection table for candidate Cox’s mixed effects models for the Kenya dataset – deaths from intentional human causes

| Model | AICc | ΔAIC |
| --- | --- | --- |
| 7 | 109.897 | 0.000 |
| 3 | 111.291 | 1.394 |
| 6 | 111.684 | 1.787 |
| 1 | 111.745 | 1.848 |
| 5 | 117.429 | 7.532 |
| 2 | 117.773 | 7.876 |
| 0 | 118.805 | 8.909 |
| 4 | 118.861 | 8.964 |

Table S5a: Candidate Cox Proportional Hazards models for the Kenya dataset – deaths from predation, conspecific and injury

| Model | Group Size | Temperature (90) | Rainfall (30) | Temperature:Rainfall |
| --- | --- | --- | --- | --- |
| 0 | NULL | |  |  |
| 1 |  | x |  |  |
| 2 | x |  |  |  |
| 3 | x | x |  |  |
| 4 |  |  | x |  |
| 5 | x |  | x |  |
| 6 |  | x | x |  |
| 7 |  | x | x | x |

Table S5b: Model selection table for candidate Cox’s mixed effects models for the Kenya dataset – deaths from predation, conspecific and injury

| Model | AICc | ΔAIC |
| --- | --- | --- |
| 2 | 125.647 | 0.000 |
| 3 | 127.373 | 1.726 |
| 5 | 127.378 | 1.732 |
| 0 | 127.709 | 2.063 |
| 4 | 129.308 | 3.661 |
| 1 | 129.660 | 4.013 |
| 7 | 131.007 | 5.361 |
| 6 | 131.219 | 5.572 |

Table S6a: Candidate Cox Proportional Hazards models for the Kenya dataset – deaths from disease

| Model | Group Size | Temperature (90) | Rainfall (30) | Temperature:Rainfall |
| --- | --- | --- | --- | --- |
| 0 | NULL | |  |  |
| 1 |  | x |  |  |
| 2 | x |  |  |  |
| 3 | x | x |  |  |
| 4 |  |  | x |  |
| 5 | x |  | x |  |
| 6 |  | x | x |  |
| 7 |  | x | x | x |

Table S6b: Model selection table for candidate Cox’s mixed effects models for the Kenya dataset – deaths from disease

| Model | AICc | ΔAIC |
| --- | --- | --- |
| 1 | 107.653 | 0.000 |
| 6 | 109.328 | 1.676 |
| 3 | 109.512 | 1.859 |
| 7 | 111.295 | 3.642 |
| 0 | 114.495 | 6.842 |
| 4 | 115.870 | 8.218 |
| 2 | 116.492 | 8.839 |
| 5 | 117.851 | 10.199 |

Table S7a: Candidate Cox Proportional Hazards models for the Zimbabwe dataset – deaths from all causes

| Model | Group Size | Temperature (90) | Rainfall (30) | Temperature:Rainfall |
| --- | --- | --- | --- | --- |
| 0 | NULL | |  |  |
| 1 |  | x |  |  |
| 2 | x |  |  |  |
| 3 | x | x |  |  |
| 4 |  |  | x |  |
| 5 | x |  | x |  |
| 6 |  | x | x |  |
| 7 |  | x | x | x |

Table S7b: Model selection table for candidate Cox’s mixed effects models for the Zimbabwe dataset – deaths from all causes

| Model | AICc | ΔAIC |
| --- | --- | --- |
| 3 | 153.265 | 0.000 |
| 2 | 153.896 | 0.631 |
| 7 | 154.181 | 0.916 |
| 5 | 154.277 | 1.012 |
| 0 | 154.431 | 1.166 |
| 1 | 154.557 | 1.292 |
| 4 | 155.369 | 2.104 |
| 6 | 156.313 | 3.048 |

Table S8a: Candidate Cox Proportional Hazards models for the Zimbabwe dataset – deaths from unintentional human causes

| Model | Group Size | Temperature (90) | Rainfall (30) |
| --- | --- | --- | --- |
| 0 | NULL | |  |
| 1 | x |  |  |
| 2 |  | x |  |
| 3 |  |  | x |

Table S8b: Model selection table for candidate Cox’s mixed effects models for the Zimbabwe dataset – unintentional human causes

| Model | AICc | ΔAIC |
| --- | --- | --- |
| 0 | 55.672 | 0.000 |
| 1 | 84.423 | 28.751 |
| 2 | 85.884 | 30.212 |
| 3 | 91.845 | 36.173 |

Table S9a: Candidate Cox Proportional Hazards models for the Botswana dataset – deaths from all causes

| Model | Group Size | Temperature (30) | Rainfall (7) | Temperature:Rainfall |
| --- | --- | --- | --- | --- |
| 0 | NULL | |  |  |
| 1 |  | x |  |  |
| 2 | x |  |  |  |
| 3 | x | x |  |  |
| 4 |  |  | x |  |
| 5 | x |  | x |  |
| 6 |  | x | x |  |
| 7 |  | x | x | x |

Table S9b: Model selection table for candidate Cox’s mixed effects models for the Zimbabwe dataset – deaths from all causes

| Model | AICc | ΔAIC |
| --- | --- | --- |
| 2 | 84.423 | 0.000 |
| 7 | 85.565 | 1.142 |
| 3 | 85.884 | 1.461 |
| 0 | 90.023 | 5.599 |
| 4 | 91.595 | 7.172 |
| 1 | 91.845 | 7.422 |
| 6 | 93.488 | 9.065 |
| 5 | 95.477 | 11.054 |

Figure S1: Histogram of deaths by month across all three sites
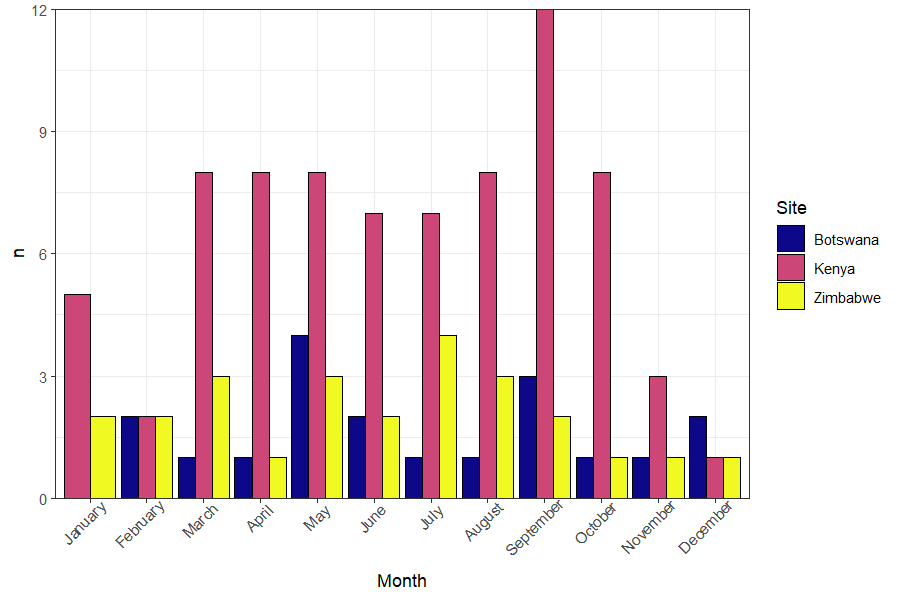


Figure S2: Raw survival curves for the three sites.


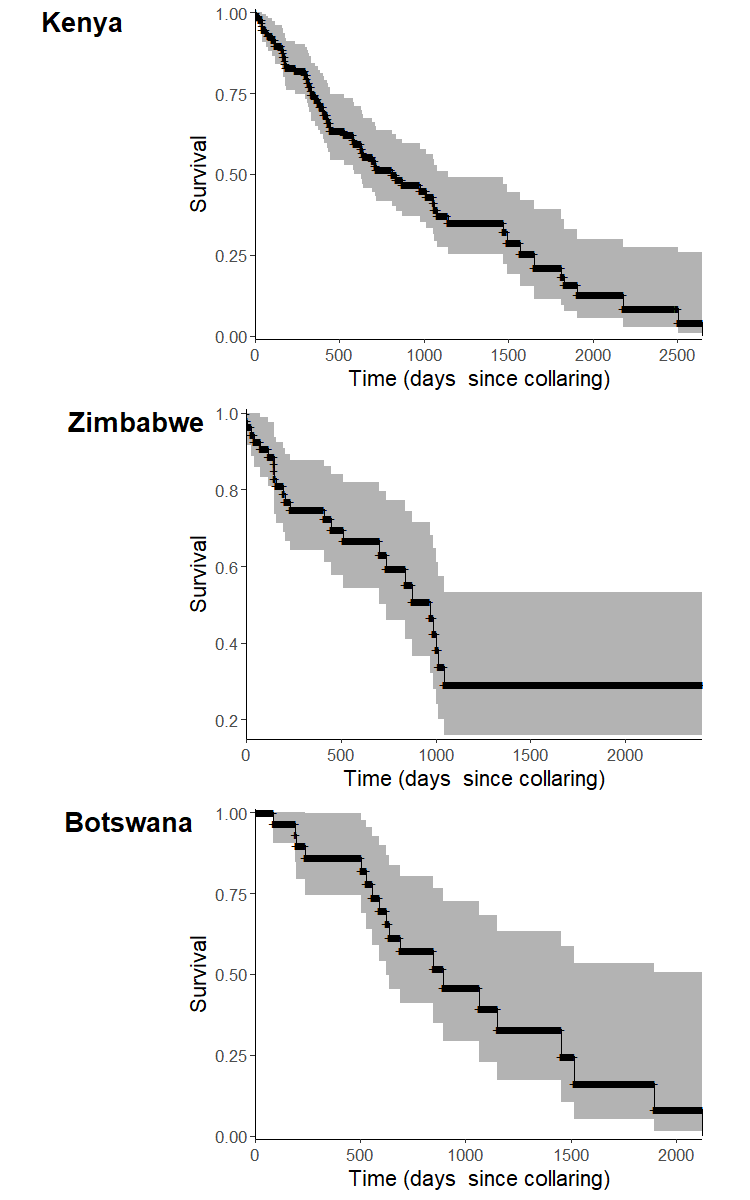

Supplement: Supplementary file 1 — Supplementary Material [file ECE3-11-8495-s001.docx]
